# Floral resources,energetic value and pesticide residues in provisions collected by *Osmia bicornis* along a gradient of oilseed rape coverage

**DOI:** 10.1038/s41598-023-39950-5

**Published:** 2023-08-17

**Authors:** Anna Misiewicz, Łukasz Mikołajczyk, Agnieszka J. Bednarska

**Affiliations:** 1 Institute of Nature Conservation, Polish Academy of Sciences, A. Mickiewicza 33, 31-120 Kraków, Poland; 2 Institute of Environmental Sciences, Jagiellonian University, Gronostajowa 7, 30-387 Kraków, Poland

**Keywords:** Agroecology, Biodiversity, Environmental monitoring, Conservation biology

## Abstract

Pollinators in agricultural landscapes are facing global decline and the main pressures include food scarcity and pesticide usage. Intensive agricultural landscapes may provide important food resources for wild pollinators via mass flowering crops. However, these are monofloral, short-term, and may contain pesticide residues. We explored how the landscape composition with a different proportion of oilseed rape (6–65%) around *Osmia bicornis* nests affects floral diversity, contamination with pesticides, and energetic value of provisions collected by this species of wild bees as food for their offspring. Altogether, the bees collected pollen from 28 plant taxa (6–15 per nest) and provisions were dominated by *Brassica napus* (6.0–54.2%, median 44.4%, 12 nests), *Quercus sp.* (1.2–19.4%, median 5.2%, 12 nests), *Ranunculus sp.* (0.4–42.7%, median 4.7%, 12 nests), Poaceae (1.2–59.9%, median 5.8%, 11 nests) and *Acer sp.* (0.6–42%, median 18.0%, 8 nests). Residues of 12 pesticides were found in provisions, with acetamiprid, azoxystrobin, boscalid, and dimethoate being the most frequently detected at concentrations up to 1.2, 198.4, 16.9 and 17.8 ng/g (median 0.3, 10.6, 11.3, 4.4 ng/g), respectively. Floral diversity and energetic value of provisions, but not the Pesticide Risk Index depended on landscape structure. Moreover, pollen diversity decreased, and energetic value increased with landscape diversity. Thus, even a structurally simple landscape may provide diverse food for *O. bicornis* if the nest is located close to a single but resource-diverse patch. Both *B. napus* and non-crop pollen were correlated with pesticide concentrations.

## Introduction

Pollinators provide essential ecosystem services for agricultural production^1^, and a third of human food production benefits directly or indirectly from insect pollination^2^. However, in recent years, insects, including bee pollinators, have been exposed to many stressors and their biomass, abundance and species richness are declining over the world^3,4^ with potentially detrimental effects on the ecosystem services they provide^5^. About 20% of pollination services in agricultural production are provided by wild bees^6^. A very important wild pollinator of various crops is the solitary bee *Osmia bicornis*^7–10^, which is often a more effective pollinator than the honeybees^11^.

A reduction in floral resource abundance and diversity observed in agroecosystems due to landscape simplification and habitat loss, together with widespread exposure to pesticides, are the main threats to pollinating insects^12^. Natural flower-rich habitats have been converted into large-scale agricultural monocultures in the last few decades^13^. Such large-scale crop monocultures are usually not attractive to pollinators due to the lack of floral resources (e.g., cereals) or, in case of mass flowering crops (e.g., oilseed rape), they provide short-lived, monofloral, and thus nutritionally unbalanced nectar and pollen resources^14–16^. Furthermore, mass-flowering crops are usually intensively treated with pesticides^12^, which may increase pollinator mortality and could reduce their efficiency^17,18^. Pesticide residues were found both in the pollen of mass-flowering crops and in wild flowers growing in the field margins^19,20^ and as many as 14 different compounds have been detected in winter *Brassica napus*^21^. *Brassica napus* is the second most essential oilseed crop and is considered the main valuable nectar-producing plant in the world^22^. However, the effect of the presence of oilseed rape around the nest on solitary bees is not fully clear. It was shown that proximity to oilseed rape crop positively affects the number of nesting *O. bicornis*, but it was suggested that oilseed rape benefits solitary bees in the form of abundant nectar for foraging flights rather than pollen for brood provisioning^23–25^. However, *B. napus* has been also identified as an important source of pollen for *O. bicornis* larvae^26–29^. The quality of pollen is very important to the larvae but, if contaminated with pesticides, it can affect negatively larval development^30^. Also the nutritional value of pollen may vary depending on the landscape^31^.

The aim of this study was to investigate the influence of agricultural landscape structure with different proportion of oilseed rape crop in the area around *O. bicornis* nests on floral diversity, the level of contamination with pesticides and the energetic value of provisions retrieved from nests established in 12 sites. We hypothesized that an increasing proportion of oilseed rape around the nests reduces landscape heterogeneity and, in consequence, the pollen diversity of larval provision. Increased pesticide exposure risk was also expected, as the diets with higher proportion of oilseed rape pollen are more likely to be contaminated with pesticides. Because the diversity of floral resources may depend on the availability of different habitats around the nest, the effect of local landscape characteristic within the circular areas of 500 m and 1000 m around the nests, which correspond to the typical foraging distances of *O. bicornis*^26,32^ was also studied. We hypothesized that landscape with lower proportion of oilseed rape crop in the area around *O. bicornis* nests and with more natural elements provides higher floral diversity, lower pesticide risk and better food quality in terms of its energy value.

## Results

### Floral diversity

**Figure 1:**
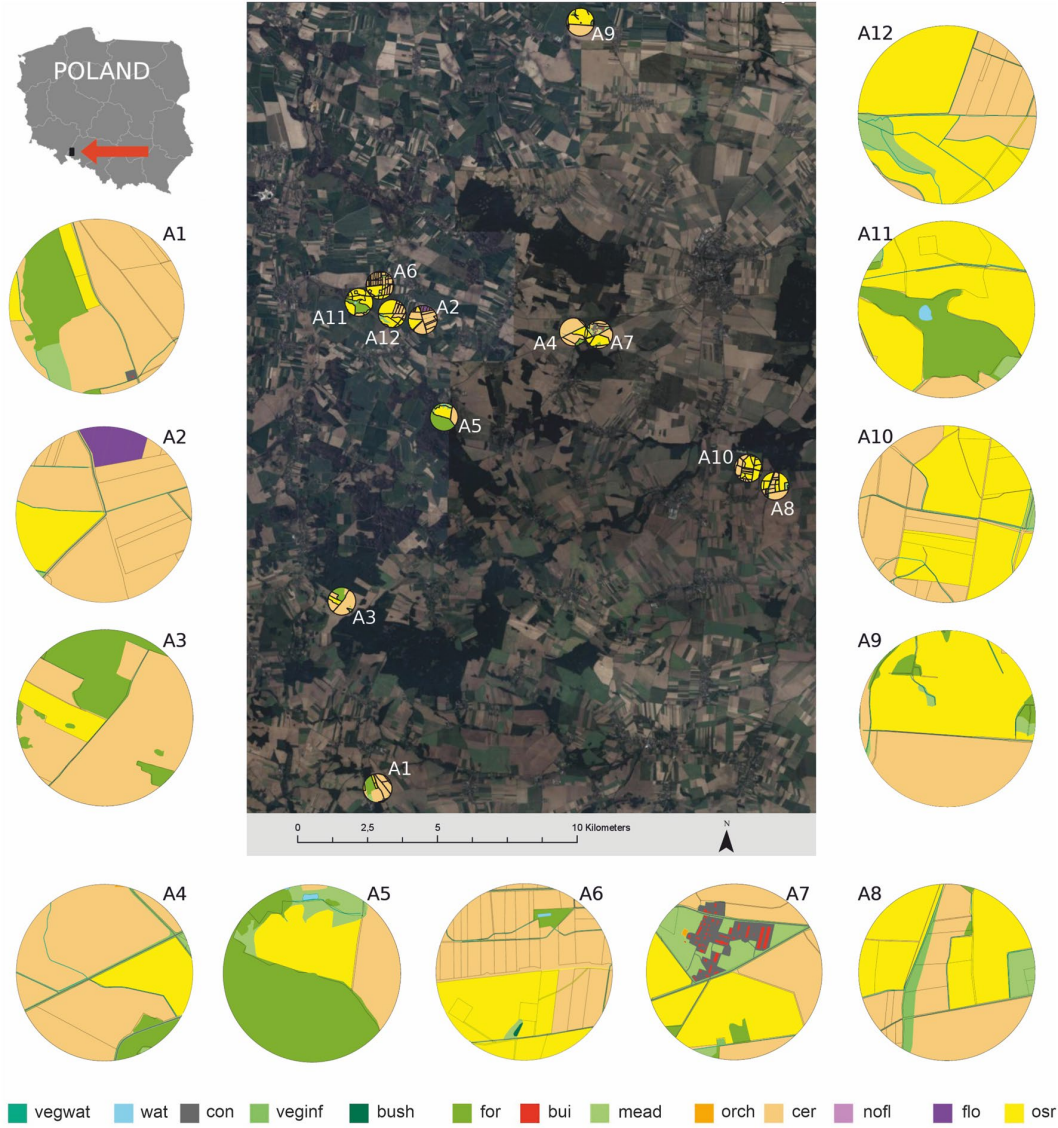
Location of the 12 study sites (A1–A12) in the agricultural landscape in the Opolskie province (Poland) and the characteristics of the 500 m buffer with the oilseed rape (yellow) and other 13 landscape elements (see Table S2 for detailed description). Map created with the use of Esri ArcMap 10 and GIMP 2.10.30 software. Satellite imagery data: Google, CNES / Airbus, Airbus Maxar Technologies obtained via Google Earth Pro software.

Altogether, the bees collected pollen from 28 plant taxa (6–15 per nest), and three of them—*Brassica napus*, *Quercus sp*. and *Ranunculus sp*.—were recorded in all 12 nests (Table 1). Provisions were dominated by *B. napus* pollen*,* which constituted 6% to 54% (median 44.4%). Poaceae (1–60%, median 5.8%), *Ranunculus sp*. (0.4–43%, median 4.7%), *Acer sp*. (0.6–42%, median 18.0%), and *Quercus sp*. (1–19%, median 5.2%) prevailed upon the rest pollen types, but up to 13 plant taxa contributed less than 1% to the diet of *O. bicornis* (Table 1). Pollen floral diversity (expressed as PENS) decreased with increasing LDI (*p* = 0.011) and FA1 (*p* = 0.007) in 500 m buffer (Fig. S2), although the significance of the latter relationship was driven by a single nest (A7 nest located in the site with high contribution of concrete, buildings, vegetation close to infrastructure and orchards; Fig. 1). The model including both explanatory variables i.e., LDI and FA1) was significant at *p* = 0.0003, R^2^ = 84% (see Table S4 for β parameters). On a larger scale (1000 m buffer), PENS was not related to any of the four landscape variables (ORC, FA1, FA2, LDI).

**Table 1:** Mean proportion of pollen types (identified to family, genus, or species level) in bee collected provisions per nest in the twelve nests (A1–A12) located in the agricultural landscape, pollen diversity expressed as pollen effective number of species (PENS), and provision energetic value.

Pollen type	Mean proportion of plant taxa in provisions in different sites [%]												Min [%]	Max [%]	Median [%]
	A1	A2	A3	A4	A5	A6	A7	A8	A9	A10	A11	A12			
**Brassicaceae/** ***Brassica napus***	54.21	28.25	6.17	50.72	52.15	6.00	51.18	45.35	43.61	45.21	8.50	10.14	6.00	54.21	44.41
*Acer sp.*	30.74	–	28.30	18.56	17.38	–	42.31	4.74	–	4.71	0.59	–	0.59	42.31	17.79
*Achillea typ*	0.17	–	–	–	–	–	–	–	–	–	–	–	0.17	0.17	–
*Aesculus sp.*	0.17	0.41	–	–	0.19	–	0.54	–	–	–	0.79	–	0.17	0.79	0.41
Caprifoliaceae/ Lonicera	–	–	–	–	–	–	–	–	0.39	–	–	–	0.39	0.39	–
*Carex sp.*	–	–	–	–	–	–	–	0.38	–	7.90	–	3.11	0.38	7.90	3.11
Caryophyllaceae	–	0.41	0.17	0.41	–	–	–	–	–	–	–	–	0.17	0.41	0.41
*Centaurea cyanus*	–	–	–	–	–	–	–	0.19	–	–	–	–	0.19	0.19	–
Chenopodiaceae	–	–	0.17	–	–	–	–	–	–	–	–	–	0.17	0.17	–
*Cornus sp.*	–	–	–	–	–	–	–	0.38	–	–	–	–	0.38	0.38	–
*Eleagnus sp.*	–	–	–	–	–	–	–	–	–	0.34	–	–	0.39	0.34	–
*Hypericum sp.*	5.29	0.62	1.03	0.41	0.56	7.35	–	–	0.98	–	9.68	21.33	0.41	21.33	1.03
*Juglans sp.*	0.50	1.24	–	0.62	0.56	0.39	0.18	1.14	–	–	–	0.62	0.18	1.24	0.59
*Lamium sp.*	–	–	–	–	–	–	–	0.19	–	–	0.40	–	0.19	0.40	0.29
*Malus sp.*	–	–	–	–	–	–	–	–	0.79	0.50	–	–	0.50	0.79	–
*Papaver sp.*	–	0.82	0.17	–	–	0.58	–	0.38	0.20	–	–	–	0.17	0.82	0.38
*Pinus sp.*	–	–	0.17	0.21	0.19	–	–	0.19	–	0.17	0.40	–	0.17	0.40	0.19
*Plantago sp.*	0.50	1.24	–	–	–	0.39	–	–	–	0.34	0.59	1.45	0.34	1.45	0.54
Poaceae	1.16	8.87	1.20	3.71	5.79	25.73	–	17.46	2.36	5.21	59.88	27.74	1.20	59.88	5.79
*Prunus sp.*	0.83	2.27	–	0.21	0.75	1.55	–	2.28	0.59	2.69	3.36	3.73	0.21	3.73	1.91
*Pyrus sp.*	–	9.90	–	–	–	–	–	–	–	–	–	–	9.90	9.90	–
*Quercus sp.*	3.64	5.15	10.81	3.30	19.44	1.55	5.24	6.83	14.93	4.87	5.53	1.24	1.24	19.44	5.20
*Ranunculus sp.*	1.16	38.97	0.51	21.24	1.31	42.75	0.54	0.38	9.82	5.04	4.35	27.95	0.38	42.75	4.69
*Rubus sp.*	–	1.86	11.32	–	1.12	13.15	–	19.54	5.50	21.51	5.93	1.66	1.12	21.51	5.93
*Rumex sp.*	0.17	–	38.94	–	–	–	–	–	18.86	–	–	0.21	0.17	38.94	9.53
*Salix sp.*	1.49	–	0.17	–	0.19	–	–	–	0.39	0.17	–	0.62	0.17	1.49	0.29
*Trifolium repens*	–	–	0.86	0.62	0.37	0.58	–	0.57	1.57	0.67	–	0.21	0.21	1.57	0.60
*Viola tricolor*	–	–	–	–	–	–	–	–	–	0.67	–	–	0.67	0.67	–
**PENS**	3.54	5.51	4.98	3.88	4.00	4.82	2.53	4.94	5.32	5.68	4.32	5.92			
**Provision energetic value [kJ/g]**	18.62	18.02	17.74	18.32	18.77	17.58	17.15	18.18	18.19	17.46	18.08	17.41

### Pesticide residues

Altogether, residues of 12 pesticides (eight fungicides, three insecticides, and one herbicide), 1–9 per nest, were detected in *O. bicornis* provisions at concentrations ranging from 0.11 ng/g (for acetamiprid) to 198.40 ng/g (for azoxystrobin). Acetamiprid was detected in 9 out of 12 nests and azoxystrobin, boscalid, and dimethoate were the next most frequently detected pesticides (7 out of 12 nests, Table 2).

**Table 2:** Concentrations of active substances [ng/g] detected in provisions collected by *Osmia bicornis* bees as food for their larvae in twelve nests located in the agricultural landscape.

Active substance	Type^a^	Concentrations of active substances [ng/g] detected in provisions in different nests												LOQ [ng/g]^b^	Number of nests detected	Percent of nests detected [%]	Min [ng/g]^c^	Max [ng/g]^c^	Median [ng/g]	Oral 48-h LD_50_ for honeybee [µg/bee]^d^
		A1	A2	A3	A4	A5	A6	A7	A8	A9	A10	A11	A12							
Acetamiprid	I	–	0.37	–	0.83	–	0.28	0.33	0.12	0.11	1.16	0.11	0.22	0.10	9	75.00	0.11	1.16	0.28	14.53
Azoxystrobin	F	–	22.55	10.60	–	–	8.95	5.50	8.90	12.00	198.40	–	–	1.00	7	58.30	5.50	198.40	10.60	> 25.00
Boscalid	F	15.45	10.20	15.75	–	16.95	11.25	–	–	–	–	7.90	5.90	0.01	7	58.30	5.90	16.95	11.25	100.00
Chlorothalonil	F	–	–	–	–	–	2.15	–	–	–	–	–	–	1.00	1	8.30	2.15	2.15	–	> 40.00
Difenoconazole	F	–	–	–	–	–	–	6.55	–	–	40.30	–	–	0.01	2	16.70	6.55	40.30	23.43	> 177.00
Fluopyram	F, N	–	4.93	–	41.10	–	2.88	12.23	–	–	–	10.03	2.08	1.00	6	50.00	2.08	41.10	7.48	> 102.30
Fluxapyroxad	F	–	2.05	–	–	–	2.75	–	–	–	–	2.40	6.55	1.00	4	33.30	2.05	6.55	2.58	> 110.90
Picoxystrobin	F	3.70	–	–	–	–	–	–	–	–	–	–	–	0.10	1	8.30	3.70	3.70	–	> 200.00
Tebuconazole	F	24.35	–	16.20	–	–	–	10.45	18.55	–	6.30	–	–	1.00	5	41.70	6.30	24.35	16.20	> 83.05
Dimethoate	I, A	–	2.75	–	12.05	–	7.85	4.35	17.08	–	–	1.70	3.00	1.00	7	58.30	1.70	17.08	4.35	0.10
Omethoate	I, A	–	1.13	–	6.33	–	4.98	1.40	9.13	–	–	–	1.33	5.00	6	50.00	1.13	9.13	3.19	0.05
Prosulfocarb	H	–	–	–	–	–	26.40	–	–	–	–	–	–	1.00	1	8.30	26.40	26.40	–	103.40
**Provision mass [mg]**^e^		246.50	241.80	239.80	233.20	252.20	216.30	238.90	241.30	212.20	254.30	229.50	231.90							
**Pesticide Risk Index**^f^		0.01 × 10^–3^	1.26 × 10^–3^	0.02 × 10^–3^	5.89 × 10^–3^	0.004 × 10^–3^	3.96 × 10^–3^	1.75 × 10^–3^	8.72 × 10^–3^	0.01 × 10^–3^	0.21 × 10^–3^	0.40 × 10^–3^	1.34 × 10^–3^							

Twelve active substances were detected above their limits of quantification (LOQ^b^), including 8 fungicides, 3 insecticides and 1 herbicide, and were used to calculate pesticide risk index with toxic unit (TU) approach based on 48-h LD_50_ values for orally exposed adult honeybee; ‘– ‘ means not detected.

^a^I—Insecticide, H—Herbicide, F—Fungicide, N—Nematicide, A—Acaricide.

^b^LOQ values for the 510 active substances tested can be found in Table S7 of the Supplementary materials in Bednarska et al.^26^. Four concentrations of omethoate were detected below the LOQ.

^c^Min and max given for detected active substance.

^d^Because neither larval nor adult LD_50_ values specific to *Osmia sp*. were available, the oral acute 48-h LD_50_ values for adult honeybees were used to calculate pesticide Risk Index. Oral acute 48-h LD_50_ values for adult honeybees available from the Pesticide Properties Database (https://sitem.herts.ac.uk/aeru/ppdb/en/Reports/321.htm).

^e^Provision mass expressed as the average provision mass in the nest calculated based on data for the two types of cells: the most inner cells in the nesting cavities (i.e., those in which females would most likely develop) and the outermost cells located at the posterior end of the nesting cavity (i.e., those in which males would most likely develop) (*data not published).*

^f^Pesticide Risk Index calculated for all detected active substances based on oral acute 48-h LD_50_ values for adult honeybee using Toxic Unit approach. To overcome the problem that in several cases the value for oral 48-h LD_50_ was expressed as > x, we used exactly the x value which is a conservative approach.

**Figure 2:**
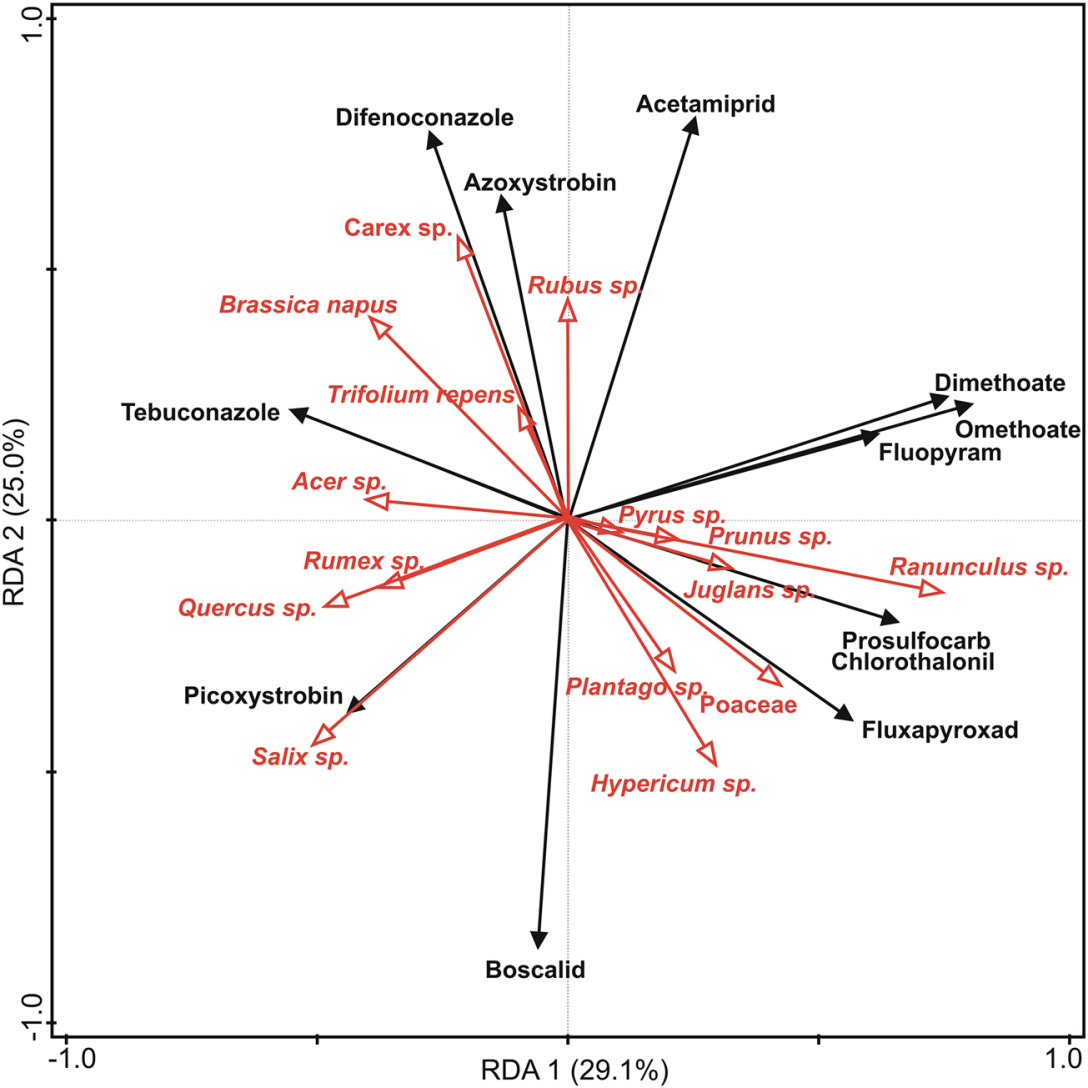
Results of a Redundancy Analysis (RDA) performed on the concentrations of pesticides in pollen, and the proprtion of pollen from the dominant taxa (i.e., more than 1% in at least one nest) in the studied 12 nests of *Osmia bicornis* in the agricultural landscape. Positions of the vectors of dependent variables on the two first RDA axes are shown by black arrows and that of the independent variables (proportion of pollen taxa) are shown by red arrows. The first ordination axis explained 29.1% of the variance of the dependent variables and the second 25.0%.

No effect of any studied landscape variables (ORC, FA1, FA2, LDI) on the Pesticide Risk Index, either for 500 m or 1000 m buffer was found. Negative relationship of the Pesticide Risk Index with PENS (RMA regression, *p* = 0.01, Fig. S3) was found, however, although significant, the percent of explained variance was negligible (R^2^ = 0.2%). No relationship between the proportion of *B. napus* in pollen and Pesticide Risk Index (simple regression, *p* = 0.6) was found. RDA showed that the presence of both *B. napus* and non-crop pollen types are correlated with the concentrations of different pesticides (Fig. 2); the first ordination axis explained 29.1% of variance of the dependent variables and the second ordination axis explained 25% of variance. High correlation between plant taxa and pesticide was found especially for *Salix sp*. and picoxystorbin, Poaceae and fluxapyroxad, as well as *Carex sp*. and difenoconazole (Fig. 2).

### Energetic value of provisions

The energetic value of provisions ranged from 17.15 kJ/g in the A7 nest to 18.77 kJ/g in the A5 nest (Table 1). A positive relationship was found between the energetic value of the provisions with the LDI (*p* = 0.003) and negative with FA1 (*p* = 0.011) (Fig. S4) for the 500 m buffer; the model including these two explanatory variables was significant at *p* = 0.008, R^2^ = 66% (see Table S4 for β parameters). However, as in case of PENS, the relationship between energetic value of provisions and FA1 was mainly driven by A7 nest, which scored high on FA1 axis (Fig. S4). A highly significant negative relationship was observed between the energetic value and PENS (RMA regression, *p* = 0.009; Fig. S5), but, the model explained only 3% of the variability.

## Discussion

The bees collected from 6 to 54% of *B. napus* pollen in the agricultural landscape with different proportion of oilseed rape around their nests. Teper and Biliński^29^, who also studied *O. bicornis* pollen provisions during the flowering period of oilseed rape, found on average 46% of oilseed rape pollen and *Brassicaceae* was indicated as a one of the main sources of *O. bicornis* pollen by Haider et al.^27^ and Peters et al.^28^. Albeit, even on sites dominated by oilseed rape (i.e., with ORC ≥ 40%), bees collected pollen from non-crop herbaceous plants (e.g., *Ranunculus sp*. at 43% in the A6 nest) and trees (mainly *Quercus sp*. and *Acer sp*. at 15% and 42% in the A7 nest, respectively), as mentioned by previous studies^33–36^. Studies by Coudrain et al.^23^ conducted in agricultural areas with different percentage of forest around nests, showed a high proportion of *Ranunculus sp*. (58.6%) and *Quercus sp*. (23.4%) among 41 pollen types found in provisions of *O. bicornis*. In our study, *Quercus sp*. pollen was collected at relatively high proportion (1.2–19.4%) even by bees whose nests were adjacent to a field of oilseed rape. This confirms previous observations that oak trees are a substantial source of pollen for *O. bicornis*^26,37,38^ and that bees can fly large distances (up to 800 m from the nest, which is close to the maximum foraging distance of the red mason bee^32^), to reach oak pollen^26^. *O. bicornis* mixes different types of pollen to ensure constant protein content of provision^39^, but Radmacher and Strom^36^ suggested that because wind-pollinated oak trees offer large amounts of pollen, *O. bicornis* females may temporarily (and locally) specialize in one or two plant species with high pollen availability to maximize the collected pollen mass per unit time.

Saunders et al.^40^ found that bees visit ca. 100 wind-pollinated plant genera, and large part of visitation records were for grasses and sedges (Poales). Schulze-Albuquerque et al.^41^ indicated that the floral cues, colour, and scent of Poaceae can attract insects. For example, honeybees, bumblebees and sweat bees (*Lasioglossum spp*.) foraged on a pasture grass from Poaceae family^42^ and Poaceae was one of the dominant pollen type (4–12%) in the diet of the Australian bee *Tetragonula carbonaria*^43^. In our study, Poaceae pollen was found in 11 out of 12 nests and accounted for up to 60% of pollen provisions (Table 1). The presence of Poaceae, but at smaller proportions (0.3–4.7%), in the *O. bicornis* diet was also confirmed by Splitt et al.^44^.

Floral diversity of pollen provisions decreased on sites with a greater share of “urban landscape” features (i.e., buildings, concrete and infrastructure, vegetation by infrastructure, orchards) and increased on sites with a higher share of vegetation close to water bodies and borders between fields and natural habitats. The significance of this relationship was driven mostly by a single nest located close to build-up area with a high share of buildings, concreate, and in-between vegetation as well as orchards. This result shows the imporatnce of more natural landscape elements for the floral diversity of wild bee collcetd pollen, however, a previous study perfomerd in different agricultural landscpaes that used similar landscape elements, showed an increase in pollen diversity with higher proportion of built-up areas around the nest^26^. Moreover, while in Factor Analysis all 14 landscape elements were included, the LDI included only those 7 landscape characteristics that are expected to be functionally relevant for the red mason bee (Table S1) and still a negative relationship with PENS was found. This may indicate that even a homogeneous landscape always contains some portion of semi-natural habitats that provide food diversity, which was also suggested by Malagnini et al.^45^ in their study of diversity of pollen collected by honeybees in an agricultural area. Malagnini et al.^45^ expressed the landscape heterogeneity around honeybee nests by both landscape composition through Principal Component Analysis and landscape diversity through Shannon diversity index (based on data for 24 land-use classes (elements). Comparably to our study, the authors expected to find highly diverse landscapes offering a wider range of pollen types in comparison to homogeneous landscapes. However, honeybees collected highly diverse pollen regardless of the landscape diversity, while landscape composition affected pollen diversity only at the end of the flowering season when the proportion of semi-natural areas started to play important role^45^. Also, the study by Danner et al.^46^ on honeybees found that pollen composition was not affected by landscape composition expressed via the Shannon diversity index. These results question the validity of using landscape diversity indices calculated based on the type of the element and its coverage to describe landscape diversity available for bees artificially introduced into the environment^45,47^. Unlike local populations, bees artificially introduced to the field together with nesting material for one season, are not constrained by nest availability, and their reproductive success mainly depends only on the degree to which a landscape facilitates or impedes access to the resource patch(es) and/or movement of bees among resource patches (nectar and pollen) (i.e., connectivity). In this case, even the existence of a single element (patch) in a small proportion (e.g., only a small multi-species flowering meadow which may contain an average 60 plant species^48^) can provide a more diverse food source than several elements (patches), which provide little diverse food (e.g., single-species strip of trees or shrubbery, monoculture of flowering crop, single-species orchard, etc.). On the other hand, LDI based on Shannon diversity index will not capture the diversity of the multi-species flower meadow, as it will treat it as a single element (patch) functionally relevant for bees. Because LDI calculated for sites dominated by a single element (patch) will be lower than for sites with several bee-relevant elements, it may produce results opposite to the expected increase in pollen diversity (PENS) with landscape diversity (LDI). Shannon diversity index quantifies the heterogeneity of landscapes, considering both richness and evenness of land-use elements (patch types), with low values of the index indicating a low landscape heterogeneity, but it does not consider species richness of the individual elements themselves. Therefore, although the widely used Shannon index has been recommended for landscape management within an ecological framework, description and interpretation of the relationships between pollen diversity (but also other variables) and Shannon-based landscape diversity indices^49^, should be made with caution. On a larger scale (1000 m buffer), our results show that floral diversity of pollen was not related to any of the four landscape variables studied. This emphasizes the importance of the local landscape, (i.e., the area in the close vicinity of the nest) for the food resources of *O. bicornis*.

Bees may be frequently exposed to different classes of pesticides through nectar, pollen, and guttation droplets^21,50–52^. We found residues of 12 pesticides in *O. bicornis* provisions with acetamiprid being the most frequently detected and dimethoate and omethoate presenting the highest risk to bees (i.e., their contribution to the pesticide risk index was the largest). The reported concentrations of acetamiprid residues detected in pollen directly collected from plants were in the range 0.02–0.82 ng/g^19,53^, similar to what we found in provisions collected by *O. bicornis* in this study (up to 0.83 ng/g, median 0.28 ng/g) and in the earlier study by Bednarska et al.^26^: 0.1–2.23 ng/g (median 0.30 ng/g). Acetamiprid belongs to neonicotinoids, which are the most widely used insecticides in the world^54^. It was proven that acetamiprid has a negative effect on adult honeybees and stingless bees, including a significantly reduced lifespan and affected locomotor activity^55,56^. In case of *O. bicornis*, Mokkapati et al.^30^ showed that although acetamiprid did not affect larval survival and larval body mass, the length of larval stage (i.e., time to cocoon formation) was significantly shorter in larvae exposed to acetamiprid compared to controls. The negative effect of other pesticides detected in our pollen samples, such as picoxystrobin and dimethoate was also confirmed in studies on adult honeybees fed ad libitum sucrose solutions containing different concentrations of these insecticides^57–59^.

Our results showed that bees are exposed to a wide spectrum of pesticides in agricultural landscapes, as previously indicated in honeybee studies^50,60^. In contrast to honeybee pollen, the one collected by solitary bees in the agricultural landscape was less frequently evaluated for pesticide residues. Bednarska et al.^26^ detected residues of 34 pesticides (with acetamiprid among the 10 found most often) in provisions collected by *O. bicornis* over the entire season in the intensively used agricultural landscape in Poland. Also, Rundlöf et al.^61^ found residues of 12 pesticides in provisions collected by *O. lignaria*, which experienced similar pesticide risk at sites without and with flower strips used to mitigate the effects of bee pesticide exposure and support bee reproduction in intensively farmed landscapes in Sweden. Centrella et al.^62^ found 28 pesticides (13 insecticides and 15 fungicides) in pollen collected by *O. cornifrons* in apple orchards and indicated that the presence of agricultural habitats within 2 km was associated with an increased level of pesticide risk. In our study, there was no effect of any of the evaluated landscape variables on the Pesticide Risk Index, including both 500 m and 1000 m buffer.

Mass-flowering crops, such as oilseed rape, are often intensively treated with pesticides^21,63^. Zioga et al.^21^ indicated up to 14 different compounds in winter *B. napus*, and the median concentrations of these compounds found in cultivated plants was higher than those in wild plants. Similarly, in individual oilseed rape pollen samples collected in China, Wen et al.^63^ found residues of at least 10 pesticides, and 4 samples contained up to 40 pesticides. It is not clear whether the pesticide residues found in our study came from contaminated oilseed rape flowers, other non-focal crops, wildflowers along field margins, or other sources, since the pesticide analysis could be performed only on mixed pollen from multiple provisions from each nest. Although no relationship between the proportion of *B. napus* in pollen and Pesticide Risk Index was found, we cannot exclude that even a small amounts of crop pollen collected by bees can lead to significant pesticide risks^15^. Moreover, we observed negative relationship between PENS and Pesticide Risk Index, showing that reduced pollen diversity (PENS) increases pesticide risk in bee collected pollen. However, this relationship explained only a small percentage of the total variance and seems to be driven by high contribution of dimethoate and omethoate to the Pesticide Risk Index. The presence of these two active substances cannot be directly linked to any specific pollen species and, in fact, the RDA analysis showed that the presence of both *B. napus* and non-crop pollen types are correlated with the concentrations of different pesticides. Contrary to our results, a positive relationship between pollen diversity and insecticide risk levels in *O. bicornis* pollen was found by Bednarska et al.^26^ which may also suggest the contamination of plants in non-crop areas.

The energetic values of provisions were similar to those estimated for honeybee pollen (16.6–17.1 kJ/g) collected by beekeepers in Portugal^64^. The energetic value of pollen increased with increasing landscpe diversity and on sites with a higher share of vegetation close to water bodies and borders between fields and natural habitats and decreased on sites with a higher share of "urban landscape" features, namely was the lowest on A7 site. Surprisingly, the provision of nest A7, although located close to "build-up areas", was dominated by theree pollen types (*B. napus* > *Acer sp.* > *Quercus sp*), was the least diverse (had the lowest value of PENS) and had the lowest caloric value.

Despite the low percentage of explained variance, the negative relationship between PENS and energetic value may show that a more diverse pollen provision does not necessairly show better quality in terms of caloric value. It should be noted, that bomb calorimetry does not necessarily correspond to digestible energy, as it measures total energetic value of a sample, including also poorly digestible parts of the pollen grain, such as the pollen wall^65^. Furthermore, the presence and number of pores of germination has been hypothesized to influence pollen digestibility^65^. At the same time, provisions taken from *O. bicornis* brood cells may contain nectar sugars, which also contribute to the caloric value of provisions, but no study has specifically determined the ratios of pollen to nectar in *O. bicornis* provisions and the factors that control that ratio. Maddocks and Paulus^66^ suggested that *O. bicornis* provision brood cells with pollen and a comparatively low proportion of nectar (2%), but in a study performed on the larvae of the alfalfa leaf-cutting bee, *Megachile rotundata* (which belongs to the same Megachilidae family as *O. bicornis*), Cane et al.^67^ estimated that the provisions consist of pollen and nectar at a 1:2 ratio (~ 33% alfalafa pollen and 67% nectar). *Brassica napus* pollen has a high energy value (12.6 kJ/g) and a high fat content (5.47%)^68^, so even a small proportion of oilseed rape pollen might influence mean calorific value of larval provision. Nevertheless, it is unlikely that proportion of *B. napus* determines the energetic value of the nest provisions in our study, as a proportion of *B. napus* in the nest with the lowest caloric value of the provision (51% in A7 nest) was similar to that of nests with the highest caloric values (52%, 54% and 50% in nests A5, A1 and A4, respectively).

## Conclusions

In conclusion, although *O. bicornis* is a generalist species, we confirmed that it prefers a certain set of plants, including trees and shrubs, if available. The pollen collected by *O. bicornis* was dominated by five taxa, including *B. napus*, by the fields of which, nests were placed during its flowering period. Both floral diversity and energy value of provisions were related to the landscape structure. The influence of the landscape structure and diversity was visible on a small scale (500 m buffer) only, which is in line with the rather small foraging radius of that species^32,69^. However, caution is needed for the interpretation of the results based on relationships with the Shannon-based landscape diversity index, as it does not consider species diversity within individual landscape elements (cover types). In our study, the presence of landscape elements, their sizes (i.e., proportions in the landscape) and connectivity between different landscape elements were captured by the scores for both FA1 and F2, but the results for relationship of PENS with LDI indicate that it is still necessary to include the quality (e.g., diversity) of landscape elements themselves. Although time-consuming, measures based on provisions collected by bees for their offspring rather than landscape characteristics provide a complete picture of food resources in an agricultural landscape. After all, it is what the bees have collected, regardless of where they collected it, that determines the survival and development of the offspring in the nest. We showed that bee larvae are exposed through their food to a variety of pesticides, the concentrations of which are correlated with both crop pollen (*B. napus*) and other non-crop plants (e.g., *Ranunculs sp*., Poaceae, *Carex sp*.). Although both the mass-flowering crops and the nearby flowers and trees can be contaminated with a wide range of pesticides, in the studied landscape the pesticide risk generally decreased with increasing floral diversity of provisions. Thus, introduction of varied flora into the agricultural landscape should be considered in pollinator conservation and management decisions to mitigate the effects of agricultural landscape.

## Methods

### Sites and landscape characteristic

Data were collected during oilseed rape blooming season in 2019 from twelve sites located in the agricultural landscape of the Opolskie province, Poland (Fig. 1). The sites represented the gradient of oilseed rape coverage (ORC, 6–65%) within non-overlapping circular areas of 500 m radius (called the “buffer” thereafter) (Table S1). The local landscape structure around each nest was characterised based on land cover maps created at two spatial scales (500 m and 1000 m buffers), using 13 discrete, non-overlapping landscape elements (land cover types) and two linear features representing land fragmentation (Table S2). It was analysed in ArcMap 10^70^ as described in Misiewicz et al.^71^. Landscape elements (without oilseed rape coverage which was used separately due to its importance for bees and as a controlled experimental factor) were reduced to two factors (FA1 and FA2) using Factor Analysis, which explained respectively 32.4% and 21.0% of the total variability in local landscape characteristics in the 500 m buffer, and 29.0% and 27.1% respectively in the 1000 m buffer. FA1 for the 1000 m buffer and FA2 for the 500 m buffer captured almost the same landscape elements, which scored similarly: “arable lands” features (i.e., cereals, nonflowering and flowering crops but also bushes and the length of borders between fields) scored high, while “landscape naturalness” (meadows, forests, and the length of borders between fields and natural habitats) scored low on those axes. However, FA2 for 1000 m buffer and FA1 for 500 m buffer were inversed: in general, landscape elements characteristic for “urban areas” (concreate, buildings, but also vegetation close to infrastructure) that scored high on FA1 for 500 m buffer, scored low on FA2 for 1000 m and, at the same time, those scored low on FA1 for 500 m (water and vegetation by water) scored high for FA2 for 1000 m. Only some elements (e.g., orchards) shifted their position in FA1 and FA2 factors, scoring higher either in “built-up areas” (FA1, buffer 500 m) or “arable lands” (FA1, buffer 1000 m). See Misiewicz et al.^71^ for more details on Factor Analysis. In addition, the Landscape Diversity Index (LDI, i.e., Shannon-Wiener index) of seven landscape elements that present potential foraging habitats for bees (i.e., vegetation by water, vegetation by infrastructure, bushes, forests, meadows, orchards, flowering crops; Table S2) was calculated for each study site.

### Solitary bees and experimental design

One artificial nest (Fig. S1A) with 16 nesting cases providing 360 nest cavities, and ca. 550 commercially available cocoons of *O. bicornis* (Pszczelinka, Kapka Sp. z. o.o., Poland) were placed on the perimeters of the oilseed rape field in each site centre. The nests were left in the field from 17th April to 4th June 2019. In agricultural landscapes, flower resources and pesticide use change over space and time^72^. Thus, flower phenology influences bee activity and expected pesticide exposure^73^. To ensure the availability of food resources in the close vicinity from the nest, we allowed females to gather food for their larvae only during the restricted period of oilseed rape blooming.

Upon transferring to the laboratory, half of each nest (8 upper nesting cases) was kept under changing temperature conditions to breed the next generation of bees as described by Misiewicz et al.^71^ and the second half (8 lower nesting cases) was frozen at − 20°C for pollen provision sampling (Fig. S1B). The samples of provisions were kept in the freezer for 4 months before used for chemical analysis.

Eggs or larvae were removed from the brood cells and the pollen provision from a separate nesting cavity was placed in a separate Eppendorf tube and stored at − 20°C until further analysis. For this study, only nesting cavities with less than six provisions per cavity were used (27–104 cavities per nest; Table S3); the remaining cavities were used for another study. The pollen provisions were thoroughly mixed to create a combined representative sample for the entire nest. Each combined sample was divided into three subsamples used for palynological analysis (~ 3 g), pesticide analysis (~ 30 g) and to determine pollen energetic values (~ 0.4 g).

### Palynological analysis

The palynological analysis was performed using microscope slides, following the method described in the Supplementary Materials. *Brassica napus*, *Centaurea cyanus*, *Trifolium repens,* and *Viola tricolor* were identified at the species level and other taxa at the genus (20) or family level (4). All pollen types for each nest site are presented in Table 1. The pollen effective number of species (PENS)^26^ was calculated for each nest as exp(H’), where H’ is the Shannon-Wiener diversity index^74,75^.

### Pesticide analysis

For pesticide analysis, pollen samples were screened for residues of 510 different active substances using LC–MS/MS or GC–MS/MS techniques at the Institute of Plant Protection, National Research Institute, Laboratory of Food and Feed Safety, Białystok, Poland (see Bednarska et al*.*^26^ for all details on chemical analysis, including multiresidues and single methods used, LC–MS/MS and GC–MS/MS parameters and validation parameters for 510 active substances analyzed (LOQ levels and recovery (%)). The results were reported as the mean value of two parallel determinations for each nest and a site Pesticide Risk Index was calculated using toxic unit (TU) approach as described by Bednarska et al.^26^ to capture the combined hazard and exposure level to multiple substances at a site. In short, the TU for each nest was calculated as the sum of the products of the concentration of each active substance and the mean provision mass per larvae divided by the oral LD_50_ of that active substance for adult honeybees (Table 2), using a following equation:

$$\mathrm{Pesticide\, Risk\, Index}=\sum \frac{\mathrm{Acive\, substance}\left[\frac{ng}{g}\right] \times \mathrm{provision}\, \mathrm{mass}[g]}{{\mathrm{LD}}_{50}\frac{ng}{\mathrm{bee}}}$$

The mean provision mass still available for larvae in each nest was calculated from the provision mass collected from those nesting cavities which contained six or more brood cells and were used for another study (see Table 2).

### Determination of pollen energetic values

The energetic value of the vacuum-dried provision samples was measured with a Semimicro Calorimeter (model 6725) containing a calorimeter thermometer (model 6772) and a Semimicro Oxygen Bomb (model 1109A) (Parr Instrument Company). The energetic value of the pollen in each nest was measured in 3 replicates and expressed in kJ/g dry mass (Table 1).

### Statistical analysis

For each response variable (PENS, Pesticide Risk Index, energetic value), multiple regression analyses with all landscape variables (i.e., ORC, FA1, FA2, and LDI) as explanatory variables were performed separately for 500 m and 1000 m buffers. Landscape variables were standardised, and the stepwise backward selection process was used to remove nonsignificant variables from the model so that only variables significant at *p* ≤ 0.05 remained. The normal distribution of residuals was tested for each model using the Shapiro–Wilk test.

The relationship between pollen diversity (PENS) and Pesticide Risk Index (TU) was analysed using reduced major axis (RMA) regression to test whether reduced pollen diversity increases pesticide risk in bee-collected pollen. The RMA was also used to test relationship between PENS and the energetic value of provision. The RMA was used instead of standard least-squares regression to handle errors in both the x and y variables.

Because we hypothesized that prevalence of oilseed rape pollen in the provisions would increase pesticide risk due to pesticide applications on oilseed rape fields, we tested whether the Pesticide Risk Index depends on the proportion of *B. napus* pollen found in the provisions by using a simple regression analysis. Moreover, a redundancy analysis (RDA) with Monte Carlo test with 499 unrestricted permutations was performed to determine the pattern of variability in pesticides concentrations among sites by the proportion of plant taxa as explanatory variables. For RDA we selected pollen contributing more than 5% to the diets in any nest (i.e., 13 pollen types were not included in this analysis).

Multiple regression analyses and simple regression analyses were performed using Statgraphics Centurion 18 (StatPoint, Herndon, VA, USA; http://www.statgraphics.com), RMA regression was performed using PAST 3 software for Windows (https://softfamous.com/past/) and RDA analysis was performed in Canoco ver. 5^76^.

### Supplementary Information

Supplementary Information.

## Data Availability

The raw data are available from the corresponding author upon request.
