# Supplementary material for: Floral resources,energetic value and pesticide residues in provisions collected by Osmia bicornis along a gradient of oilseed rape coverage
Source: Sci Rep. 2023 Aug 17;13:13372. doi: 10.1038/s41598-023-39950-5 (PMC10435552; doi:10.1038/s41598-023-39950-5)
Supplement: Supplementary file 1 — Supplementary Information. [file 41598_2023_39950_MOESM1_ESM.docx]

**SUPPLEMENTARY MATERIALS**

Floral resources, energetic value and pesticide residues in provisions collected by *Osmia bicornis* along a gradient of oilseed rape coverage

Anna Misiewicz^1*^, Łukasz Mikołajczyk^1^,^2^, Agnieszka J. Bednarska^1^

^1^Institute of Nature Conservation, Polish Academy of Sciences, A. Mickiewicza 33, 31-120 Kraków, Poland

^2^Institute of Environmental Sciences, Jagiellonian University, Gronostajowa 7, 30-387 Kraków, Poland

^*^[misiewicz@iop.krakow.pl](mailto:misiewicz@iop.krakow.pl)

**Methods**

**1. Palynological analysis**

For palynological analysis, 3 g of composite pollen sample from each pooled collection was taken and mixed with 100 ml of distilled water and vortexed for several times, each time for 2 minutes, over the course of 6 hours. The resulting solution was used to prepare microscope slides^1^, two slides per sample. To determine the share of pollen representing different taxa, ca. 300 grains per slide were counted^2^ along two lines chosen randomly across the cover slip at a magnification of 400x (Olympus BX41) using reference specimens and published reference collections. *Brassica napus* (oilseed rape), *Centaurea cyanus*, *Trifolium repens,* and *Viola tricolor* were identified at the species level and other taxa to the genus or family level. Average sum of pollen grains from two analyzes was calculated for each taxon and the data were expressed as the percentage content of individual type of pollen.

**2. Pesticide analysis**

The pesticide analyses were performed using the protocols and methodology fully described in Supplementary materials in **Bednarska *et al*.**^3^.

**Tables**

**Table S1.** Characteristics of the study sites in the close vicinity of *Osmia bicornis* nests (i.e., within 500 m and 1000 m radius around each nest, called “buffers”) in the agricultural landscape selected for the field study in 2019; ORC – oilseed rape coverage [%], LDI – Landscape Diversity Index.

| **Nest ID** | **Agricultural area [%] within 5x5 km around the nest** | **Natural area [%] within 5x5 km around the nest** | **ORC [%] in 500 m buffer** | **ORC [%] in 1000 m buffer** | **LDI in 500 m buffer** | **LDI in 1000 m buffer** |
| --- | --- | --- | --- | --- | --- | --- |
|  |  |  |  |  |  |  |
| A1 | 88 | 10 | 6.35 | 6.73 | 3.22 | 3.20 |
| A2 | 91 | 12 | 13.93 | 11.83 | 2.24 | 3.07 |
| A3 | 57 | 44 | 14.33 | 8.20 | 1.94 | 1.86 |
| A4 | 81 | 21 | 14.98 | 22.93 | 2.53 | 3.14 |
| A5 | 71 | 31 | 22.48 | 10.77 | 2.55 | 2.80 |
| A6 | 93 | 11 | 39.56 | 42.87 | 1.69 | 2.09 |
| A7 | 84 | 17 | 39.98 | 20.26 | 2.53 | 3.24 |
| A8 | 80 | 21 | 45.73 | 22.62 | 1.66 | 3.08 |
| A9 | 94 | 6 | 52.85 | 28.16 | 1.56 | 2.09 |
| A10 | 81 | 20 | 57.33 | 30.15 | 1.21 | 2.94 |
| A11 | 91 | 15 | 63.23 | 35.27 | 2.13 | 2.79 |
| A12 | 92 | 12 | 65.30 | 39.02 | 1.52 | 2.47 |

**Table S2.** Description of landscape characteristics (elements) used for characterization of buffers around each *Osmia bicornis* nest as described in Mikołajczyk *et al*.^4^. The landscape elements (cover types) used to calculate Landscape Diversity Index (LDI) are in boldface.

| **Landscape unit** | **Name** | **Acronym** | **Description** |
| --- | --- | --- | --- |
| **1** | **Vegetation by water bodies** | **vegwat** | **Encompasses (an arbitrarily chosen if not already mapped) 2 m wide strip of terrain that surrounds lakes, ponds, and runs along both sides o streams, brooks, rivers, drainage ditches and hydro-technical channels of a different sort. Because of its peripheral location, this type of vegetation is rarely maintained or cut and seems to remain in a relatively untouched state throughout the whole year.** |
| 2 | Water bodies | wat | Groups all bodies of water, flowing and standing, of natural and anthropogenic origin. |
| 3 | Concrete, asphalt, infrastructure | con | Groups all anthropogenically paved terrains – roads, walkways, paved yards, and other infrastructural objects like pylons, wind turbines, and transmission towers. |
| **4** | **Vegetation by infrastructure** | **veginf** | **Groups vegetation around roads, walkways, yards, and infrastructural objects. This vegetation is often maintained is some way (roads) but might as well stay forsaken (back yards).** |
| **5** | **Bushes** | **bush** | **Groups terrains covered with perennial plants, bushes, shrubs, overgrown uncultivated lands but not yet forests. Group gathers also urban parks and cemeteries.** |
| **6** | **Forests** | **for** | **Groups terrains covered with trees and underbrush.** |
| 7 | Buildings | bui | Gathers man-made structures of habitual or industrial character – edifices, houses, factories, warehouses, etc. |
| **8** | **Meadows** | **mea** | **Groups terrains covered by grasslands and meadows, offering an abundance of flowering plants when not maintained.** |
| **9** | **Orchards** | **orch** | **Groups terrains with perennial, flowering fruits plantations.** |
| 10 | Cereals | cer | Gathers agricultural land with anemophilous grasses cultivated for grain. Terrains prone to agricultural treatment (e.g., insecticide spraying). |
| 11 | Non-flowering crops | noflo | Groups agricultural land with crops not producing regular flowers or harvested before blooming. Terrains prone to agricultural treatment. |
| **12** | **Flowering crops** | **flo** | **Gathers agricultural land with crops producing flowers. Terrains prone to agricultural treatment.** |
| 13 | Oilseed rape | oil | Groups agricultural land planted with intensively flowering oilseed rape (*Brassica napus*). Prone to heavy agricultural treatment. |
| 14 | Field-to-field borders | ff | Counts the total length of field boundaries and is used as a proxy for average agricultural plot size and land fragmentation. |
| 15 | Field-to-natural borders | fn | Counts the total length of boundaries between agricultural land and natural (or semi-natural) habitats and is used as a proxy for potential shelter availability for arthropods in an agricultural landscape. |

**Table S3.** Number of nesting cavities per nest of *Osmia bicornis* used to prepare mixed provision samples for palynological analysis, screening of active substances and energetic value measurements.

| **Nest ID** | **No. of total nesting cavities with provisions** | **No. of nesting cavities with provisions selected for analysis in this study** |
| --- | --- | --- |
| A1 | 124 | 104 |
| A2 | 76 | 40 |
| A3 | 100 | 60 |
| A4 | 74 | 38 |
| A5 | 75 | 64 |
| A6 | 106 | 68 |
| A7 | 60 | 30 |
| A8 | 104 | 62 |
| A9 | 47 | 27 |
| A10 | 60 | 38 |
| A11 | 147 | 73 |
| A12 | 138 | 64 |
| **Sum** | **1111** | **668** |

**Table S4.** Results of the backward stepwise multiple regression analysis for 500 m buffer to describe the relationship between explanatory variables, i.e., Pollen Effective Number of Species (PENS), Pesticide Risk Index and energetic value of pollen and four independent variables (oilseed rape coverage (ORC, %), FA1, FA2 and Landscape Diversity Index (LDI)). The regression parameters b and β (the latter for the model on standardized variables) and p values are reported only for the variables included in the final model containing only significant explanatory variables (at p ≤ 0.05); p, R^2^, R^2^_adj_ – values for the final model. *NS* – not significant.

| Explanatory variable |  | Independent variable | | | | | | |
| --- | --- | --- | --- | --- | --- | --- | --- | --- |
|  |  | ORC | FA1 | FA2 | LDI | p | R^2^ | R^2^_adj_ |
| PENS | p | ─ | 0.007 | ─ | 0.011 |  |  |  |
|  | b | ─ | -0.121 | ─ | -0.867 | 0.0003 | 83.8% | 80.2% |
|  | β | ─ | -0.548 | ─ | -0.499 |  |  |  |
| Pesticide Risk Index | p | ─ | ─ | ─ | ─ | *NS* | ─ | ─ |
|  | b |  |  |  |  |  |  |  |
|  | β |  |  |  |  |  |  |  |
| Energetic value | p | ─ | 0.011 | ─ | 0.003 |  |  |  |
|  | b | ─ | 781.370 | ─ | -79.790 | 0.0078 | 66.0% | 58.4% |
|  | β | ─ | -361.730 | ─ | 449.480 |  |  |  |

**Figures**


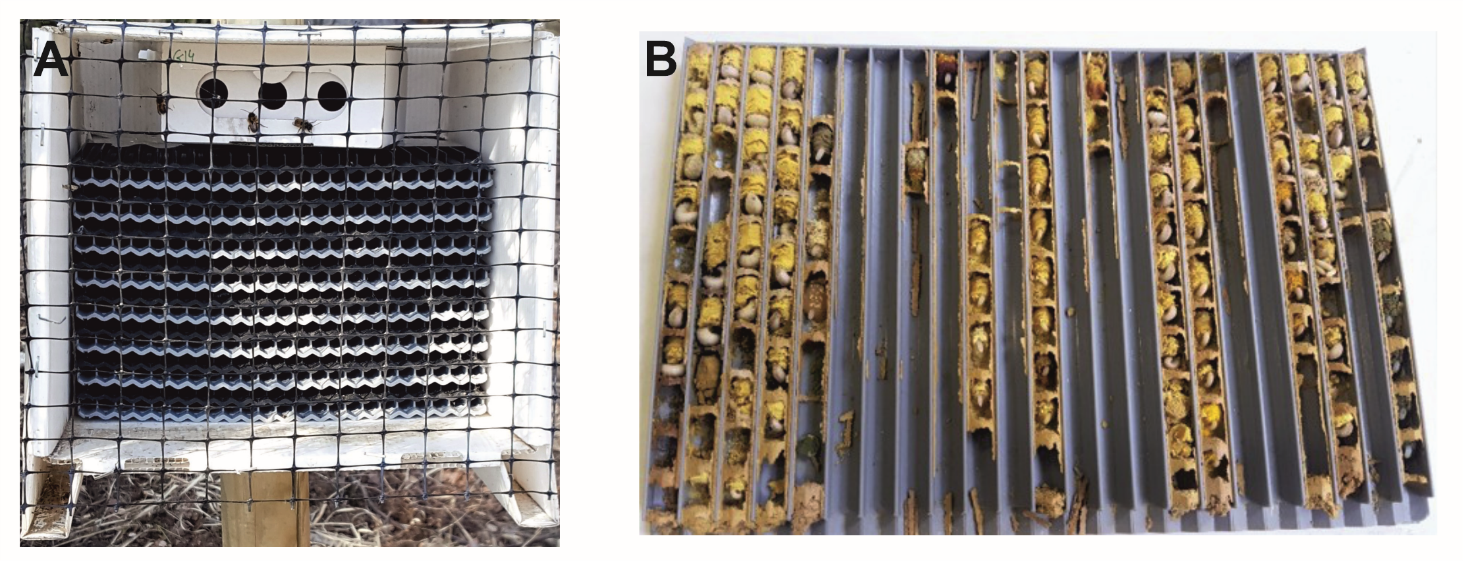


**Figure S1.** (A) The artificial nest made of 16 polystyrene elements (nesting cases), stacked on top of each other. Nesting cases were placed in the box-shaped housing made of durable and weather-resistant polypropylene together with a carton box with *Osmia bicornis* cocooned adults. To protect solitary bees against birds or rodents, each nest was closed with a plastic grid (1×1cm) and attached to a wooden pole at a height of ca. 1 m above the ground. (B) Example of a nesting case with pollen provisions stored.


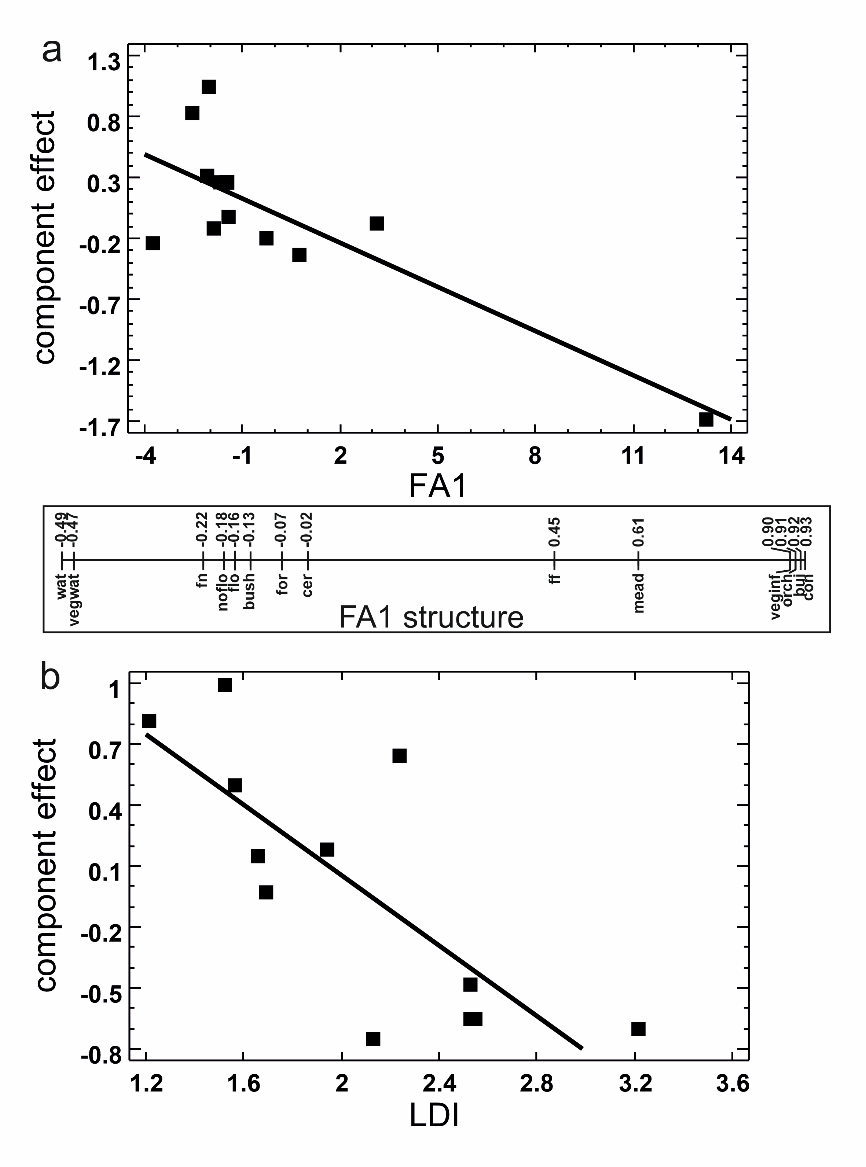


**Figure S2.** Results of the multiple regression analysis for the 500 m buffer: negative effects of (A) FA1 (p = 0.007) and (B) LDI (p = 0.011) on the pollen diversity (PENS). The overall model including both variables was significant at p ≤ 0.001 and explained 83.8% of the variability. The line shows the relative change in the predicted values of the PENS when changing (A) FA1 or (B) LDI over their observed ranges. Each point (site) is then plotted by adding its residuals to a line. Note that the values on the y-axis are the residuals of the part of the model explained by another significant variable. The right side of the graph A shows the variables scores for 14 landscape elements that describe sites (*see Table S2 for a full description of the landscape units*) spread on the unitless FA1 axis.


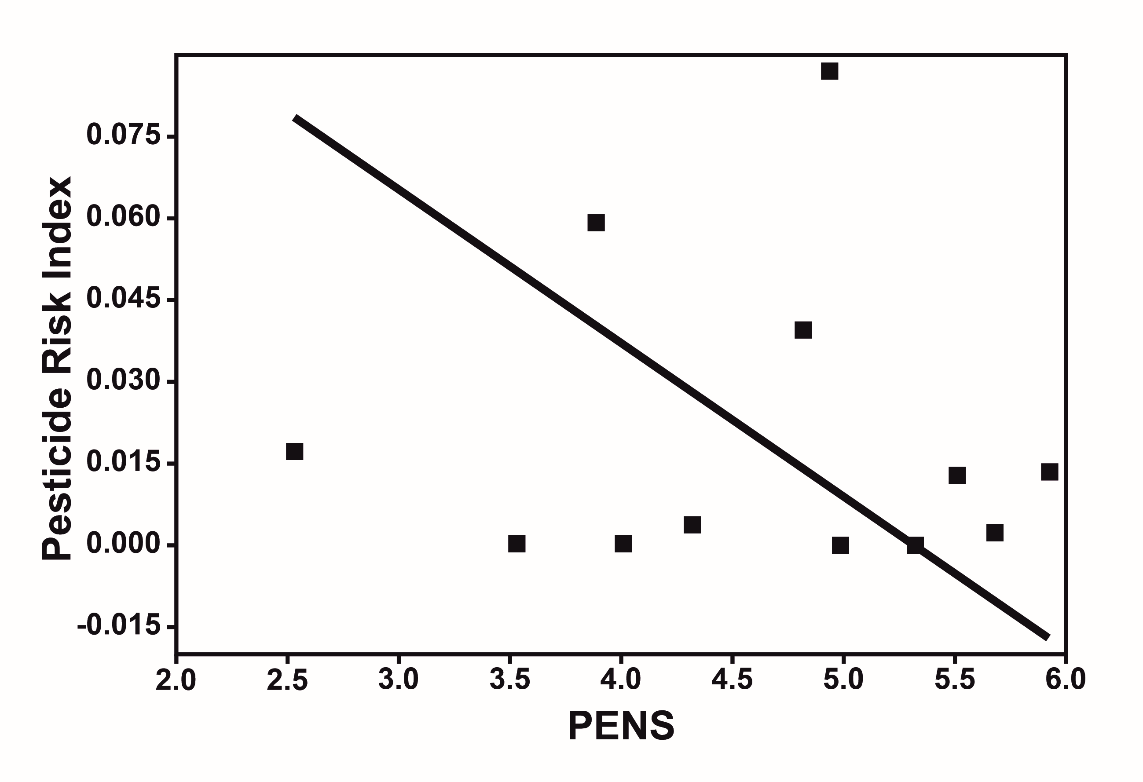


**Figure S3.** Results of the reduced major axis (RMA) regression: negative relationship between pollen diversity expressed as pollen effective number of species (PENS) and Pesticide Risk Index expressed as toxic unit (p = 0.01) analysed in pollen provisions collected by *Osmia bicornis* for their larvae in 12 nests located in agricultural landscape.


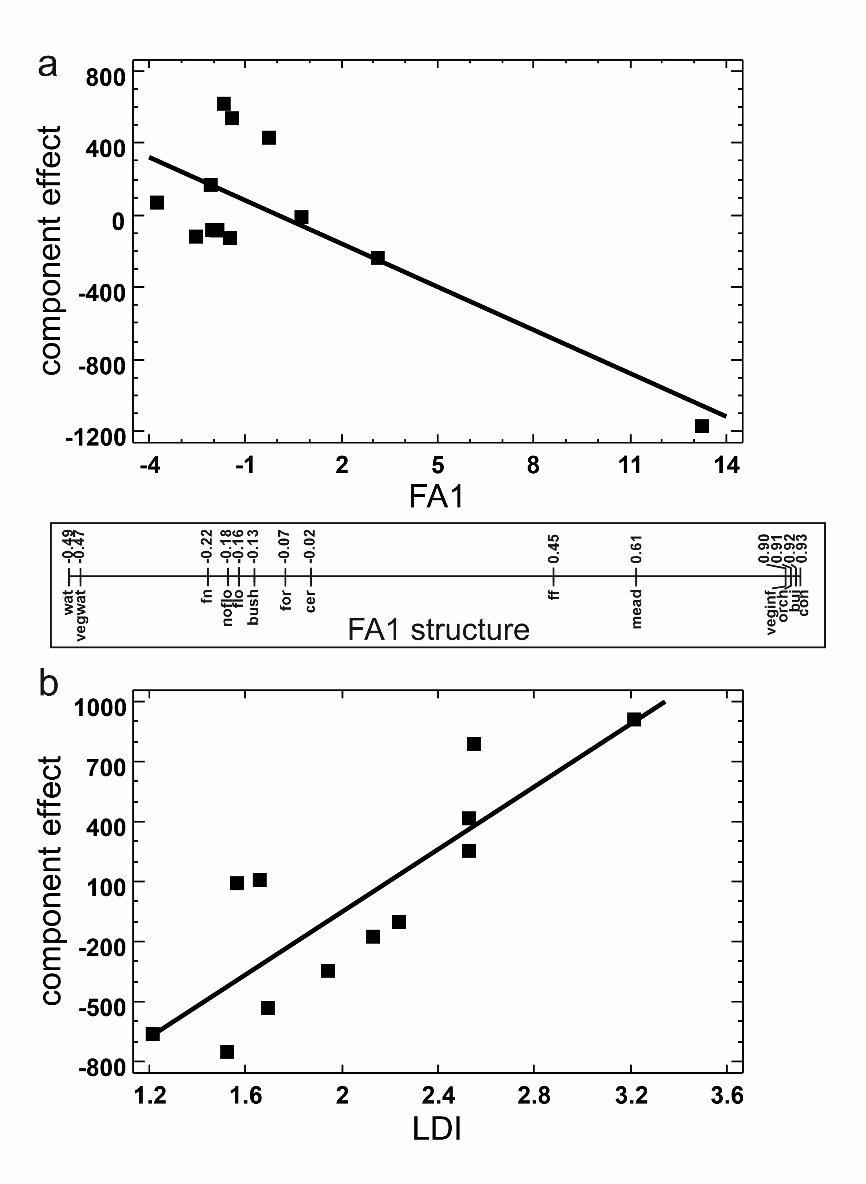


**Figure S4.** Results of the multiple regression analysis for the 500 m buffer: negative effects of (A) FA1 (p = 0.011) and positive effect of (B) LDI ( p = 0.003) on the energetic value of pollen. The overall model including both variables was significant at p = 0.008 and explained 66% of the variability. The line shows the relative change in the predicted values of the energetic value of pollen when changing (A) FA1 or (B) LDI over their observed ranges. Each point (site) is then plotted by adding its residuals to a line. Note that the values on the y-axis are the residuals of the part of the model explained by another significant variable. The right side of the graph A shows the variables scores for 14 landscape units describing sites (*see Table S2 for a full description of the landscape units*) spread on the unitless FA1 axis.


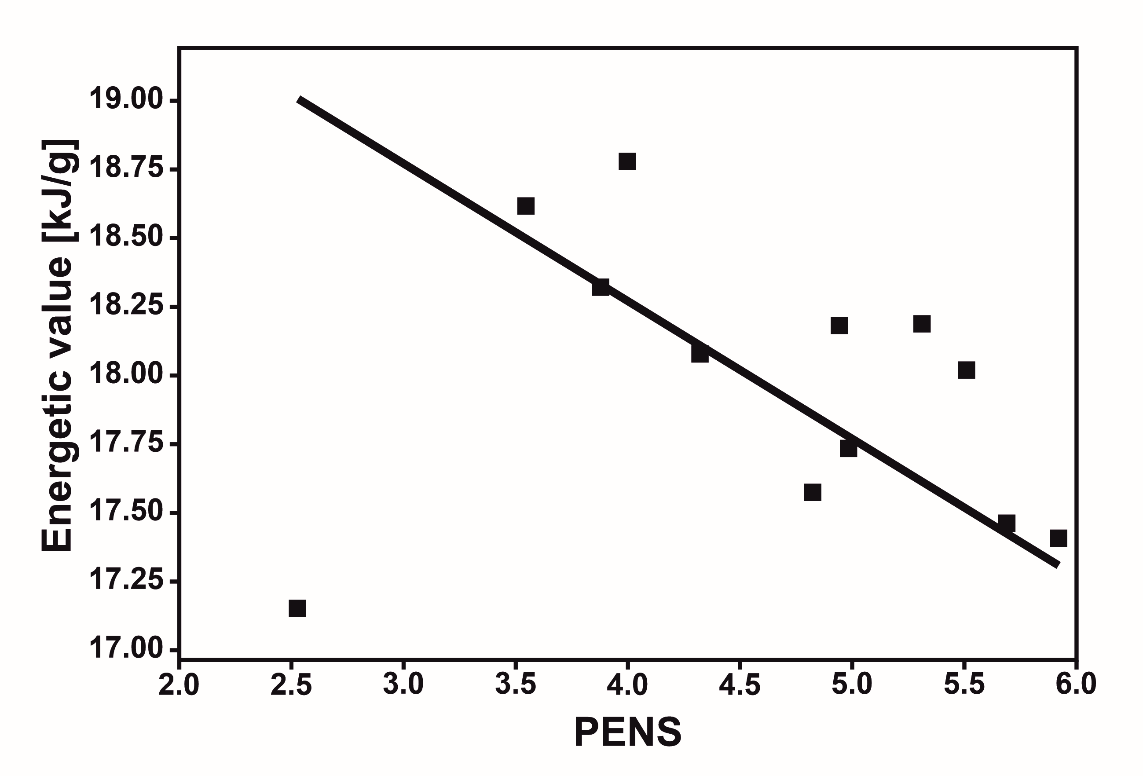


**Figure S5.** Results of the reduced major axis (RMA) regression: negative relationship between pollen diversity expressed as pollen effective number of species (PENS) and energetic value of pollen (p = 0.009) analysed in pollen provisions collected by *Osmia bicornis* for their larvae in 12 nests located in agricultural landscape.

**References**

1. Sawyer, R.W. Pollen identification for beekeepers. Ed. R. S. Pickard, University College Cardiff Press (1981).
2. Moar, N. T. Pollen analysis of New Zealand honey. *New Zealand J. Agric. Res.* **28**, 39–70. <https://doi.org/10.1080/00288233.1985.10426997> (1985).
3. Bednarska, A. J. *et al*. Effects of agricultural landscape structure, insecticide residues, and pollen diversity on the life-history traits of the red mason bee *Osmia bicornis*. *Sci Total Environ.* **809**, 151142. <https://doi.org/10.1016/j.scitotenv.2021.151142> (2022).
4. Mikołajczyk, Ł., Laskowski, R., Ziółkowska, E. & Bednarska, A. J. Species-specific landscape characterisation method in agro-ecosystems. *Ecol indic.* **129**, 107894. <https://doi.org/10.1016/j.ecolind.2021.107894> (2021).
